# Use of Electronic Clinical Data to Track Incidence and Mortality for SARS-CoV-2–Associated Sepsis

**DOI:** 10.1001/jamanetworkopen.2023.35728

**Published:** 2023-09-29

**Authors:** Claire N. Shappell, Michael Klompas, Christina Chan, Tom Chen, Sanjat Kanjilal, Caroline McKenna, Chanu Rhee

**Affiliations:** 1 Department of Population Medicine, Harvard Medical School/Harvard Pilgrim Health Care Institute, Boston, Massachusetts; 2 Division of Pulmonary and Critical Care Medicine, Department of Medicine, Brigham and Women’s Hospital, Boston, Massachusetts; 3 Division of Infectious Diseases, Department of Medicine, Brigham and Women’s Hospital, Boston, Massachusetts

## Abstract

**Importance:**

Efforts to quantify the burden of SARS-CoV-2–associated sepsis have been limited by inconsistent definitions and underrecognition of viral sepsis.

**Objective:**

To describe the incidence and outcomes of SARS-CoV-2–associated sepsis vs presumed bacterial sepsis using objective electronic clinical criteria.

**Design, Setting, and Participants:**

This retrospective cohort study included adults hospitalized at 5 Massachusetts hospitals between March 2020 and November 2022.

**Exposures:**

SARS-CoV-2–associated sepsis was defined as a positive SARS-CoV-2 polymerase chain reaction test and concurrent organ dysfunction (ie, oxygen support above simple nasal cannula, vasopressors, elevated lactate level, rise in creatine or bilirubin level, and/or decline in platelets). Presumed bacterial sepsis was defined by modified US Centers for Disease Control and Prevention adult sepsis event criteria (ie, blood culture order, sustained treatment with antibiotics, and organ dysfunction using identical thresholds as for SARS-CoV-2–associated sepsis).

**Main Outcomes and Measures:**

Trends in the quarterly incidence (ie, proportion of hospitalizations) and in-hospital mortality for SARS-CoV-2–associated and presumed bacterial sepsis were assessed using negative binomial and logistic regression models.

**Results:**

This study included 431 017 hospital encounters from 261 595 individuals (mean [SD] age 57.9 [19.8] years, 241 131 (55.9%) females, 286 397 [66.5%] from academic hospital site). Of these encounters, 23 276 (5.4%) were from SARS-CoV-2, 6558 (1.5%) had SARS-CoV-2–associated sepsis, and 30 604 patients (7.1%) had presumed bacterial sepsis without SARS-CoV-2 infection. Crude in-hospital mortality for SARS-CoV-2–associated sepsis declined from 490 of 1469 (33.4%) in the first quarter to 67 of 450 (14.9%) in the last (adjusted odds ratio [aOR], 0.88 [95% CI, 0.85-0.90] per quarter). Crude mortality for presumed bacterial sepsis was 4451 of 30 604 patients (14.5%) and stable across quarters (aOR, 1.00 [95% CI, 0.99-1.01]). Medical record reviews of 200 SARS-CoV-2–positive hospitalizations confirmed electronic health record (EHR)–based SARS-CoV-2–associated sepsis criteria performed well relative to sepsis-3 criteria (90.6% [95% CI, 80.7%-96.5%] sensitivity; 91.2% [95% CI, 85.1%-95.4%] specificity).

**Conclusions and Relevance:**

In this retrospective cohort study of hospitalized adults, SARS-CoV-2 accounted for approximately 1 in 6 cases of sepsis during the first 33 months of the COVID-19 pandemic. In-hospital mortality rates for SARS-CoV-2–associated sepsis were high but declined over time and ultimately were similar to presumed bacterial sepsis. These findings highlight the high burden of SARS-CoV-2–associated sepsis and demonstrate the utility of EHR-based algorithms to conduct surveillance for viral and bacterial sepsis.

## Key Points

**Question:**

How did the frequency and mortality for SARS-CoV-2–associated sepsis differ from presumed bacterial sepsis during the COVID-19 pandemic?

**Findings:**

In this retrospective cohort study of 431 017 inpatient encounters at 5 Massachusetts hospitals between March 2020 and November 2022, SARS-CoV-2–associated sepsis was present in 1.5% of all admissions and 28.2% of SARS-CoV-2–positive hospitalizations, whereas presumed bacterial sepsis was present in 7.1% of hospitalizations. Between the first and last study quarters, SARS-CoV-2–associated sepsis mortality decreased from 33.4% to 14.9% while presumed bacterial sepsis mortality was stable at 14.5%.

**Meaning:**

These findings suggest that SARS-CoV-2–associated sepsis was common and had higher mortality than presumed bacterial sepsis early in the COVID-19 pandemic.

## Teaser

This cohort study assesses the incidence and outcomes of SARS-CoV-2–associated sepsis vs presumed bacterial sepsis by using objective electronic clinical criteria at 5 Massachusetts hospitals.

## Introduction

Sepsis is the syndrome of life-threatening organ dysfunction caused by a dysregulated host response to infection and is a major cause of death, disability, and health care spending worldwide.^1,2,3^ While consensus definitions have always been agnostic to the type of pathogen causing sepsis,^1,4,5^ many clinicians and researchers have historically equated sepsis with bacterial infections. Most epidemiologic studies of sepsis have reported extremely low proportions of viral sepsis or have not reported it at all, but this is likely a function of underrecognition and undertesting rather than the absence of viral sepsis.^6,7,8,9^

The SARS-CoV-2 pandemic and historical surges of respiratory syncytial virus (RSV) and influenza have made evident that viral infections can be important contributors to the overall burden of sepsis.^10,11^ However, rigorous data regarding the incidence and mortality of viral sepsis are lacking, limited by slow adoption and inconsistent use of the term sepsis to describe severe viral infections associated with organ dysfunction.^12,13,14,15,16^ Previous investigations specifically assessing the prevalence and outcomes for SARS-CoV-2–associated sepsis have been limited by small sample sizes, single center design, and/or heterogeneous definitions of organ dysfunction, and were conducted using data from early in the COVID-19 pandemic.^17,18^

Electronic health record (EHR)–based surveillance using clinical markers of concurrent infection and organ dysfunction is rapidly becoming the state-of-the-art method for widescale sepsis surveillance.^10,19^ This strategy allows for more objective and reproducible analyses across hospitals and has been used by the US Centers for Disease Control and Prevention (CDC) to quantify the national burden of sepsis.^20^ It is particularly well-suited to describe trends in sepsis incidence, characteristics, and outcomes when perceptions of what constitutes sepsis and clinicians’ proclivity to diagnose sepsis are changing (eg, due to campaigns to increase sepsis awareness and recognition, updates in sepsis definitions, changing reimbursement mechanisms, and the greater appreciation of a virus’s potential to cause sepsis gained during the COVID-19 pandemic).^21^ To date, EHR-based surveillance methods have not been adapted or specifically applied to sepsis associated with SARS-CoV-2 or other viruses. In this study, we developed and validated an EHR-based definition of SARS-CoV-2–associated sepsis modeled on the CDC’s adult sepsis event (ASE) criteria and applied it to a large health care system to provide rigorous estimates of the incidence, mortality, and trends for SARS-CoV-2–associated sepsis across phases of the COVID-19 pandemic and compared these with traditional presumed bacterial sepsis.

## Methods

### Study Design, Population, and Data Source

The retrospective cohort study was approved by the institutional review board of the Mass General Brigham (MGB) health care system, and informed consent was waived due to minimal risk of harm. This study followed the Strengthening the Reporting of Observational Studies in Epidemiology (STROBE) reporting guideline.

We performed a retrospective analysis using EHR data from 5 acute care hospitals in the MGB health care system, including 2 academic hospitals (Massachusetts General Hospital and Brigham and Women’s Hospital) and 3 community hospitals (Brigham and Women’s Faulkner Hospital, Newton-Wellesley Hospital, and North Shore/Salem Medical Center). We included all adults (18 years or older) who were admitted as inpatients, admitted under observation status, or who died in the emergency department (ED) between March 1, 2020, and November 30, 2022. ED deaths were included to avoid systematically excluding high acuity patients. Encounters for patients who were transferred from 1 MGB hospital to another were merged and treated as a single encounter.

### Identifying COVID-19 and Sepsis Hospitalizations

We defined COVID-19 hospitalizations as any encounter with a positive SARS-CoV-2 polymerase chain reaction (PCR) within 3 days before or during admission, or by the presence of an institutional COVID-19 EHR flag (triggered by positive PCR results, including from outside the MGB system) overlapping with the admission and present for 5 or more calendar days, including posthospitalization days if discharge or death occurred before 5 days.^22^ Encounters from patients with a positive PCR or flag start date between 31 to 90 days prior to admission were excluded from the COVID-19 hospitalization definition to minimize patients with persistently positive PCR results following resolved infections.

Among COVID-19 hospitalizations, we defined SARS-CoV-2–associated sepsis using EHR criteria adapted from the CDC’s ASE surveillance definition. CDC’s ASE definition requires evidence of presumed infection (blood culture draw and 4 or more days of antimicrobials, fewer days if patient is discharged or dies before 4 days elapse) and concurrent organ dysfunction (eSOFA, defined as initiation of vasopressors or mechanical ventilation, elevated lactate, or changes in baseline creatinine, platelets, or bilirubin).^23^ We made 4 modifications to ASE to optimize detection of SARS-CoV-2–associated sepsis (Table 1). First, we replaced ASE’s infection criteria with a positive SARS-CoV-2 PCR result or COVID-19 EHR flag. Second, we broadened eSOFA’s respiratory criteria beyond invasive ventilation alone to include any oxygen supplementation above simple nasal cannula given that many COVID-19 patients can have severe hypoxemia but not require intubation.^24,25,26^ Third, for patients meeting eSOFA based on nonrespiratory organ dysfunction criteria, we required at least minimal evidence of respiratory dysfunction (nasal cannula or more) before or on the day other eSOFA-qualifying organ dysfunction criteria were met given that COVID-19 is primarily a respiratory disease. Fourth, we broadened the window for eSOFA to −2 to 14 days relative to the first positive PCR or valid flag date given that SARS-CoV-2 may not cause severe organ dysfunction until up to 2 weeks after onset of infection.^27,28,29^ Presumed bacterial sepsis was defined using classic CDC ASE criteria for presumed infection, but we only included antibacterial agents in the definition of antimicrobials, used the same broadened eSOFA respiratory criteria above, and excluded patients who met the criteria for a COVID-19 hospitalization. Baseline values for creatinine, bilirubin, and platelets were estimated using the best values during hospitalization or prior 90 days for community-onset sepsis or the best values during the 2 days before or after the fulfillment of presumed serious infection criteria for hospital-onset sepsis.^30^

**Table 1.** Original CDC Adult Sepsis Event Definition and SARS-CoV-2–Associated Sepsis Event

Events	Original CDC adult sepsis event	SARS-CoV-2–associated sepsis event
Presumed serious infection	Blood culture obtained (regardless of result) Antibiotics starting within ±2 d of blood culture day and continued for ≥4 d (with at least 1 intravenous antibiotic), or until ≤1 d prior to death, discharge to hospice, or comfort care	Positive COVID-19 PCR within 3 d prior to admission through discharge, or institutional COVID-19 flag which overlaps ≥5 calendar days of admission (including days posthospitalization if died or discharged before 5 d) Excludes patients who meet the following criteria: positive PCR or COVID-19 flag present 31-90 d prior to admission
Acute organ dysfunction, ≥1 of the following:	Mechanical ventilation initiation^a^ Vasopressor initiation (norepinephrine, dopamine, epinephrine, phenylephrine, or vasopressin) Doubling in serum creatinine or decrease by ≥50% of estimated glomerular filtration rate relative to baseline (excluding patients with *ICD-10* code for end-stage kidney disease, N18.6)^b^ Total bilirubin ≥2.0 mg/dL and doubling from baseline^b^ Platelet count <×10^3^ cells/μL and ≥50% decline from baseline (baseline must be ≥×10^3^ cells/μL)^b^ Serum lactate ≥2.0 mmol/L	Mechanical ventilation, noninvasive positive pressure ventilation, high flow nasal cannula, oxygen-conserving nasal cannula, or oxygen facemask initiation Vasopressor initiation (norepinephrine, dopamine, epinephrine, phenylephrine, or vasopressin) Doubling in serum creatinine or decrease by ≥50% of estimated glomerular filtration rate relative to baseline (excluding patients with *ICD-10* code for end-stage kidney disease, N18.6)^b^ Total bilirubin ≥2.0 mg/dL and doubling from baseline^b^ Platelet count <×10^3^ cells/μL and ≥50% decline from baseline (baseline must be ≥×10^3^ cells/μL)^b^ Serum lactate ≥2.0 mmol/L
Timing of organ dysfunction	Within ±2 d of blood culture day	Within 14 d of the positive PCR or flag start date or admission, whichever is later
Oxygen requirement	None	Must have required oxygen supplementation (nasal cannula or higher) on the day of or before the time of first organ dysfunction during hospitalization, or mechanical ventilation within 24 h of admission

Abbreviations: CDC, US Centers for Disease Control and Prevention; *ICD-10*, *International Statistical Classification of Diseases and Related Health Problems, Tenth Revision*; PCR, polymerase chain reaction.

SI conversion factors: To convert bilirubin to μmol/L, multiply by 17.104; platelet count to ×10^9^/L, multiply by 1.0; serum lactate to milligrams per deciliter, divide by 0.111.

^a^ For the definition of presumed bacterial sepsis used in the primary analysis, the same respiratory dysfunction criteria were used as for SARS-CoV-2–associated sepsis.

^b^ Baseline laboratory values are defined differently depending on whether infection is present-on-admission (best values within prior 90 days through hospitalization) or hospital-onset (best values during the ±2-days before and after meeting infection criteria).

### Validation of SARS-CoV-2 Definition

The SARS-CoV-2–associated sepsis surveillance definition was validated by study physicians (C.S. and C.R.) by reviewing the medical records of 200 randomly selected COVID-19 hospitalizations to determine the presence or absence of sepsis using sepsis-3 criteria and to determine whether each sepsis episode was due to SARS-CoV-2, non–SARS-CoV-2 infection, or both, as previously described.^17^ Sensitivity, specificity, area under the receiver operator curve (AUROC), negative predictive value (NPV), and positive predictive value (PPV) of the EHR definition were calculated using dichotomized medical record review-adjudicated SARS-COV-2–associated sepsis as the reference standard.

### Statistical Analyses

The incidence of SARS-CoV-2 and presumed bacterial sepsis hospitalizations, defined as the percentage of total admissions, were calculated for each quarter (ie, 3-month period) beginning in March 2020 when the first SARS-CoV-2 cases were admitted to study hospitals. The association between crude in-hospital mortality and time, by quarter, was assessed using negative binomial regression to account for potential overdispersion, with associations reported as incidence rate ratios (IRR). Incidence and mortality data were also presented as splines overlapped with observed results given that quarterly trends did not consistently follow a linear pattern. Logistic regression was used to adjust for individual covariates, including patients’ demographics (age, gender, race, body mass index, hospital site), comorbidities (derived using the Agency for Healthcare Research and Quality Elixhauser method),^31^ and the presence of positive blood and/or sputum cultures for potentially pathogenic organisms within the first 2 days of hospitalization. Data on race and gender were taken from the EHR and reflected patients’ reports at the time of hospital registration. Race and ethnicity were categorized as Asian or Pacific Islander, Black, White, other (including American Indian, Hispanic or Latino, other, or 2 or more), and missing. Race and sex were included as covariates given previous association with COVID-19 and sepsis outcomes.^8,32,33^ Vaccination and prior infection status were not included as covariates in the models given the high rate of data missingness and because our primary objective was to describe observed trends in SARS-CoV-2–associated sepsis rather than elucidate the specific reasons underlying these trends. Two subanalyses were conducted to examine the association between adjusted in-hospital mortality and quarter: (1) treating time as a continuous variable, which assumes a logit-linear trend, and (2) treating quarter as a factor variable, which allows each quarter to assume its own association. Statistical significance was established for *P* < .05. Data preparation was performed using SAS version 9.4 (SAS Institute) and Stata version 17 (StataCorp), while all statistical analyses were done using R version 4.1.3 (R Project for Statistical Computing). Preplanned sensitivity analyses were performed using alternate definitions of SARS-CoV-2–associated sepsis: (1) by narrowing the window of eligible organ dysfunction from within 14 days to 7 days of positive PCR or admission; (2) by replacing a positive PCR for SARS-CoV-2 and/or presence of COVID-19 flag in the EHR as criteria for SARS-CoV-2 infection with the presence of 1 or more *International Statistical Classification of Diseases, Tenth Revision, Clinical Modification *(*ICD-10-CM*) codes for COVID-19 (U07.1 or J12.82); and third, by narrowing the respiratory eSOFA criteria to require mechanical ventilation, high flow nasal cannula, or noninvasive positive pressure ventilation.

## Results

### Study Cohort: Case Counts, Characteristics, and Outcomes

Among 431 017 hospitalizations from 261 595 individuals (mean [SD] age, 57.9 [19.8] years; 241 131 [55.9%] female; 286 397 [66.5%] patients from an academic hospital site), 23 276 patients (5.4%) met criteria for a COVID-19 hospitalization, of whom 6558 (28.2% of COVID-19 hospitalizations and 1.5% of all admissions) had SARS-CoV-2–associated sepsis. An additional 30 604 patients (7.1% of all admissions) had presumed bacterial sepsis without SARS-CoV-2 infection. The most common organ dysfunctions in both SARS-CoV-2–associated and presumed bacterial sepsis were respiratory (5391 of 6558 [82.2%] and 10 075 of 30 484 [49.7%], respectively) and elevated lactate levels (2761 of 6558 [42.1%] and 16 848 of 30 484 [55.1%], respectively). Mortality was highest for patients with SARS-CoV-2–associated sepsis who needed vasopressors (909 of 2280 [39.9%]), had elevated bilirubin levels (186 of 482 [38.4%]), or low platelets (36.4%) (eFigure 1 in Supplement 1). Crude in-hospital mortality for SARS-CoV-2–associated sepsis was 1460 of 6558 (22.3%) overall, and crude mortality for presumed bacterial sepsis was 4451 of 30 604 patients (14.5%) and stable across quarters (aOR, 1.00 [95% CI, 0.99-1.01]). During the first 2 days of each encounter, 204 of 6558 patients with SARS-CoV-2–associated sepsis (3.1%) had positive blood cultures and 412 of 6558 (6.0%) had positive sputum cultures for potentially pathogenic organisms; for presumed bacterial sepsis, 4712 of 30 484 (15.5%) had positive blood cultures and 1862 of 30 484 (5.8%) had positive sputum cultures during the first 2 days of hospitalization. Across the entire hospitalization, 441 of 6558 patients with SARS-CoV-2–associated sepsis (6.7%) had positive blood cultures and 1279 of 6558 (19.3%) had positive sputum cultures vs 5948 of 30 484 (19.5%) and 4021 of 30 484 (13.0%) for patients with presumed bacterial sepsis. Other characteristics and outcomes of patients meeting criteria for SARS-CoV-2–associated sepsis, presumed bacterial sepsis, and neither are shown in Table 2.

**Table 2.** Demographics and Clinical Characteristics of Patients with Sepsis and Nonsepsis Encounters

Category	Patients, No. (%)		
	SARS-CoV-2–associated sepsis	Presumed bacterial sepsis	Nonsepsis encounters
Overall	6558 (1.5)	30 604 (7.1)	393 855 (91.4)
Age, median (IQR), y	67 (55 to 78)	66 (54 to 76)	60 (39 to 73)
Gender, No. (%)			
Men	3784 (57.7)	16 941 (55.4)	169 138 (42.9)
Women	2774 (42.3)	13 663 (44.6)	224 717 (57.1)
Race, No. (%)			
Asian or Pacific Islander	257 (3.9)	1064 (3.5)	15 250 (3.9)
Black	740 (11.3)	2591 (8.5)	38 144 (9.7)
White	4320 (65.9)	23 897 (78.1)	295 257 (75.0)
Other^a^	867 (13.2)	2127 (7.0)	35 439 (9.0)
Missing	374 (5.7)	925 (3.0)	9765 (2.5)
BMI, median (IQR)	27.8 (23.8 to 33.1)	26.4 (22.6 to 31.3)	27.4 (23.6 to 32.0)
Comorbidities, No. (%)^b^			
Cancer	899 (13.7)	8892 (29.1)	58 258 (14.8)
Congestive heart failure	1759 (26.8)	8785 (28.7)	52 311 (133.3)
Chronic lung disease	1834 (28.0)	7971 (26.1)	74 373 (18.9)
Diabetes	2533 (38.6)	10 011 (32.7)	82 763 (21.0)
Neurologic disease	1219 (18.6)	6390 (20.9)	42 496 (10.8)
Kidney disease	1743 (26.6)	7813 (25.5)	55 514 (14.1)
Elixhauser mortality score, median (IQR)	7 (−1 to 22)	15 (0 to 30)	0 (−3 to 11)
Academic hospital, No. (%)	4407 (67.2)	22 860 (74.7)	259 130 (65.8)
Clinical characteristics			
Positive blood or sputum culture on HD 1 or 2	568 (8.7)	6218 (20.3)	4418 (1.1)
Required ICU admission	3351 (51.1)	12 999 (42.5)	26 613 (6.8)
Hospital LOS, median (IQR)	10 (6 to 19)	9 (5 to 16)	3 (1 to 5)
ICU LOS, median (IQR)	6 (3 to 16)	4 (2 to 8)	2 (1 to 3)
Discharge disposition			
Home	3078 (46.9)	16 579 (54.2)	339 222 (86.1)
In-hospital death	1460 (22.3)	4451 (14.5)	4292 (1.1)
Hospice	173 (2.6)	1667 (5.5)	4276 (1.1)
Nonacute care facility	1832 (27.9)	7881 (25.8)	45 743 (11.6)
Acute care hospital transfer (non-MGB)	15 (0.2)	26 (0.1)	322 (0.1)

Abbreviations: BMI, body mass index (calculated as weight in kilograms divided by height in meters squared); HD, hospital day; ICU, intensive care unit; LOS, length of stay; MGB, Mass General Brigham hospital.

^a^ Other includes patients with self-identified race in the electronic health record of American Indian, Hispanic or Latino, other, or 2 or more.

^b^ Comorbidities were derived using the Elixhauser index. Cancer includes solid tumor with and without metastases and lymphoma. Diabetes includes diabetes with and without complications. Neurologic disease includes movement disorders, seizures, and other neurologic conditions. Kidney disease includes moderate and severe kidney failure.

### Performance of EHR-Based SARS-CoV-2–Associated Sepsis Definition Relative to Sepsis-3 per Medical Record Review

Sepsis due solely or in significant part to SARS-CoV-2 infection was present in 64 of 200 COVID-19 hospitalizations (32.0%) randomly selected for medical record review. EHR-based SARS-CoV-2–associated sepsis criteria were 90.6% sensitive (95% CI, 80.7%-96.5%) and 91.2% specific (95% CI, 85.1%-95.4%) for SARS-CoV-2–associated sepsis confirmed by medical record review, with an AUROC of 0.91 (95% CI, 0.87-0.95). PPV of the EHR criteria was 82.9% (95% CI, 72.0%-90.8%), while NPV was 95.4% (95% CI, 90.2%-98.3%). See eTable 1 in Supplement 1 for performance of alternate definitions used in sensitivity analyses.

### Trends in Sepsis Incidence and In-Hospital Mortality

The incidence of SARS-CoV-2–associated sepsis fluctuated widely between quarters, with higher incidence associated with local community case surges corresponding to new SARS-CoV-2 variants: 1469 of 29 615 (5.0%) in quarter 1 (March to May 2020, initial wave of wild-type virus), 1111 of 35 981 (3.1%) in quarter 4 (December 2020 to February 2021, wild type/Alpha variant), and 1145 of 37 176 (3.1%) in quarter 8 (December 2021 to February 2022, Omicron variant). Between surges, the incidence dropped to less than 2%. The trend toward less SARS-CoV-2–associated sepsis over time was not significant (IRR, 0.92 [95% CI, 0.81-1.04] per quarter). The trend toward less presumed bacterial sepsis was significant, albeit attenuated (IRR, 0.98 [95% CI, 0.98-0.99] per quarter).

**Figure 1.**
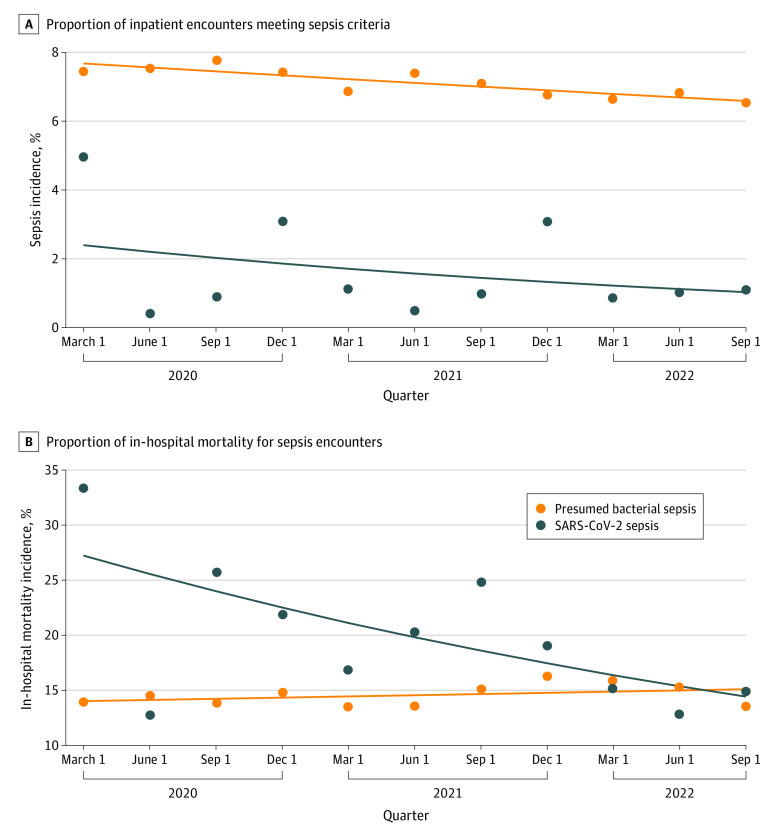
Crude Incidence and Associated Mortality by Quarter for SARS-CoV-2 and Presumed Bacterial Sepsis

Crude in-hospital mortality for SARS-CoV-2–associated sepsis declined from 490 of 1469 (33.4%) in quarter 1 (March to May 2020) to 67 of 450 (14.9%) in the last study quarter (September to November 2022) with an unadjusted quarterly IRR of 0.94 (95% CI, 0.90-0.97). Mortality associated with presumed bacterial sepsis was stable across quarters, ranging from 307 of 2204 (13.9%) in the first quarter to 362 of 2675 (13.5%) in the last quarter with an unadjusted IRR of 1.01 (95% CI, 1.00-1.02) (Figure 1). eTable 2 in Supplement 1 contains full details of incidence and mortality by quarter. Graphical representation of incidence and mortality data as splines overlapped with observed results demonstrated a nonlinear association but qualitative declines in both SARS-CoV-2–associated sepsis incidence and mortality (eFigure 2 in Supplement 1).

After adjusting for patient characteristics, in-hospital mortality decreased significantly over time for patients with SARS-CoV-2–associated sepsis (odds ratio [OR], 0.88 [95% CI, 0.86-0.90] per quarter; *P* < .001) but did not change for patients with presumed bacterial sepsis (OR, 1.00 [95% CI, 0.99-1.01] per quarter; *P* = .97). In both SARS-CoV-2–associated sepsis and presumed bacterial sepsis, age, having a body mass index (calculated as weight in kilograms divided by height in meters squared) greater than 25, kidney failure, and Elixhauser index were associated with increased sepsis mortality. Positive blood and/or sputum cultures during the first 2 days of a hospital encounter were associated with increased mortality in SARS-CoV-2–associated sepsis, while positive blood cultures were associated with lower mortality for presumed bacterial sepsis. Full model details are in Table 3.

**Table 3.** Results of Logistic Regression Model for In-Hospital Mortality in SARS-CoV-2–Associated and Presumed Bacterial Sepsis

Characteristic	SARS-CoV-2–associated sepsis		Presumed bacterial sepsis	
	Odds ratio (95% CI)	*P* value	Odds ratio (95% CI)	*P* value
Quarter				
Intercept	0.02 (0.01-0.02)	<.001	0.02 (0.02-0.03)	<.001
Continuous	0.88 (0.86-0.90)	<.001	1.00 (0.99-1.01)	.97
Hospital site				
1	1 [Reference]	NA	1 [Reference]	NA
2	0.73 (0.54-0.96)	.03	0.58 (0.47-0.70)	<.001
3	1.08 (0.91-1.28)	.37	1.09 (1.00-1.18)	.04
4	1.15 (0.93-1.42)	.18	0.74 (0.64-0.85)	<.001
5	1.05 (0.86-1.28)	.65	0.77 (0.68-0.86)	<.001
Sex				
Male	1.24 (1.09-1.43)	.002	1.01 (0.94-1.09)	.72
Female	1 [Reference]	NA	1 [Reference]	NA
Age	1.04 (1.03-1.05)	<.001	1.02 (1.02-1.02)	<.001
Race				
Asian or Pacific Islander	0.91 (0.64-1.26)	.57	1.25 (1.04-1.49)	.01
Black	1.25 (1.01-1.53)	.04	0.92 (0.80-1.04)	.19
White	1 [Reference]	NA	1 [Reference]	NA
Other	0.88 (0.70-1.09)	.23	1.02 (0.88-1.18)	.76
BMI category				
<18.5	1.08 (0.76-1.50)	.67	1.12 (0.96-1.29)	.14
18.5-24.9	1 [Reference]	NA	1 [Reference]	NA
25.0-29.9	1.31 (1.10-1.55)	.003	1.16 (1.06-1.27)	.001
30.0-34.9	1.29 (1.05-1.58)	.02	1.39 (1.25-1.55)	<.001
>35.0	2.14 (1.74-2.64)	<.001	2.20 (1.97-2.44)	<.001
Comorbidities				
Cancer	1.11 (0.91-1.36)	.28	0.91 (0.83-0.99)	.03
Diabetes	0.97 (0.84-1.12)	.68	0.89 (0.82-0.96)	.003
Chronic lung disease	1.03 (0.89-1.19)	.69	1.00 (0.92-1.08)	.99
Heart failure	0.80 (0.68-0.95)	.01	1.26 (1.65-1.37)	<.001
Hypertension	0.80 (0.67-0.95)	.01	0.80 (0.73-0.87)	<.001
Kidney failure	1.25 (1.07-1.46)	.004	1.17 (1.08-1.27)	<.001
Liver disease	1.22 (0.97-1.52)	.09	1.19 (1.08-1.31)	<.001
Elixhauser comorbidity index	1.02 (1.02-1.03)	<.001	1.03 (1.03-1.03)	<.001
Positive culture HD ≤2				
Blood	1.80 (1.28-2.53)	.001	0.78 (0.70-0.87)	<.001
Sputum	2.64 (2.01-3.47)	<.001	1.91 (1.65-2.21)	<.001

Abbreviations: BMI, body mass index (calculated as weight in kilograms divided by height in meters squared); HD, hospital day; NA, not applicable.

**Figure 2.**
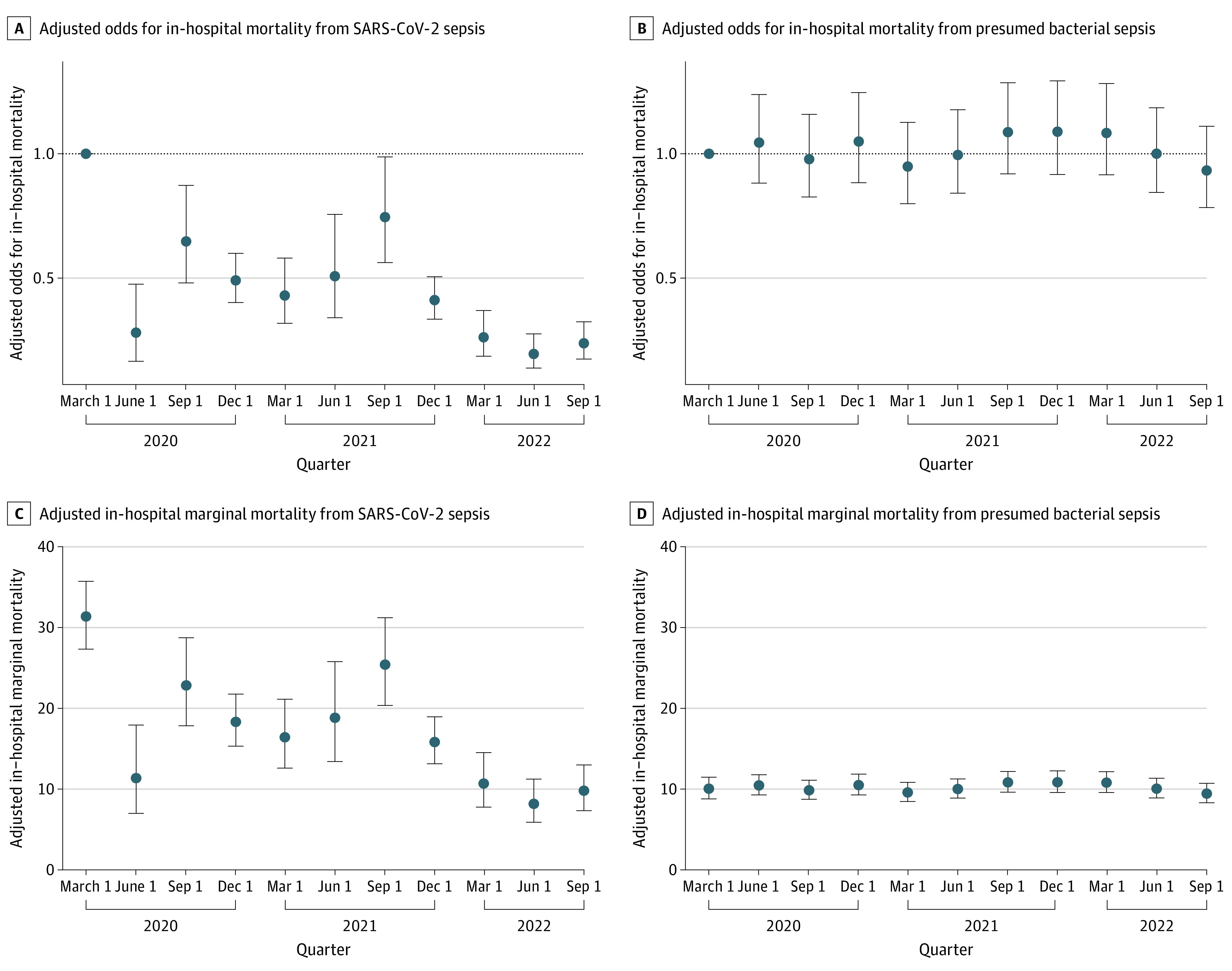
Adjusted Odds Ratios and Marginal Probabilities of Death by Quarter for SARS-CoV-2–Associated Sepsis and Presumed Bacterial Sepsis Adjusted odds ratio for in-hospital death compared with quarter 1 for patients with (A) SARS-CoV-2–associated sepsis and (B) presumed bacterial sepsis; marginal probabilities of in-hospital death by quarter for patients with (C) SARS-CoV-2–associated sepsis and (D) presumed bacterial sepsis; marginal probabilities reflect the risk of in-hospital death for a representative individual with SARS-CoV-2–associated or presumed bacterial sepsis from each quarter. Patients were most often male, White individuals, from hospital site 1, and had a body mass index (calculated as weight in kilograms divided by height in meters squared) of 18.5 to 24.9, median age, mean Elixhauser comorbidities, and culture results.

In the analysis treating quarter as a factor variable, the aORs of in-hospital death for SARS-CoV-2–associated sepsis were significantly lower in all quarters compared with quarter 1 (March to May 2020). Further, the marginal probability of death declined across quarters with the exception of quarter 2, which had an unusually low number of SARS-CoV-2 cases and deaths (eTable 2 in Supplement 1). By the end of the study period (November 2022), the mortality of SARS-CoV-2–associated sepsis approximated that of presumed bacterial sepsis (Figure 2). There were no quarters where the aOR for death for presumed bacterial sepsis differed significantly from quarter 1.

### Sensitivity Analyses

Trends in crude and adjusted in-hospital mortality for SARS-CoV-2–associated sepsis were similar using prespecified alternative definitions for SARS-CoV-2–associated sepsis. All models showed statistically significant declines in crude and adjusted mortality by quarter (eTable 3 in Supplement 1).

## Discussion

Among more than 400 000 admissions to 5 Massachusetts hospitals during the first 33 months of the COVID-19 pandemic, SARS-CoV-2–associated sepsis was present in 28.2% of patients admitted with SARS-CoV-2 infections and 1.5% of all hospital encounters. The incidence of SARS-CoV-2–associated sepsis fluctuated in tandem with changes in local community incidence but trended toward fewer cases between the pandemic onset and November 2022. In-hospital mortality rates were initially high for SARS-CoV-2–associated sepsis (33.4%) but decreased to 14.9% by November 2022. By contrast, bacterial sepsis was more common at 7.1% of hospitalizations but associated with a lower in-hospital mortality rate (14.5%) that was stable throughout the study. Our findings suggest that SARS-CoV-2 was a variable but significant contributor to the burden of sepsis in hospitalized patients during the study period.

To our knowledge, this study is the first to systematically compare the incidence and mortality for SARS-CoV-2–associated sepsis vs presumed bacterial sepsis. A meta-analysis^18^ of 151 published COVID-19 cohorts from early in the COVID-19 pandemic estimated that sepsis was present in 78% of patients with COVID-19 who were in the ICU and 33% of patients who were hospitalized with COVID-19 but were not in the ICU, using the pooled frequencies of organ dysfunction and replacement as sepsis proxies. However, this study was limited by heterogeneous definitions for sepsis and insufficient data to calculate mortality rates and focused exclusively on the initial months of the COVID-19 pandemic before vaccines, targeted therapeutics, immunomodulators, and updated respiratory management strategies were used.

Our study uses objective, EHR-based criteria adapted from the CDC’s ASE to identify SARS-CoV-2–associated sepsis, allowing accurate and consistent estimates of sepsis prevalence and mortality over time. Near universal SARS-CoV-2 testing of all patients who were hospitalized during the first years of the COVID-19 pandemic helped ensure complete case capture, which, historically, has not been possible to do for viral sepsis because of undertesting.^34^ Our surveillance definition performed well relative to sepsis-3 criteria applied via physician review, with high sensitivity and specificity for detecting sepsis secondary to SARS-CoV-2 itself rather than bacterial infection. The congruence between estimates of SARS-CoV-2–associated sepsis incidence using our EHR-based approach and previous approaches using medical record reviews and pooled organ dysfunctions provides further support for its validity.^17^

In-hospital mortality was higher for SARS-CoV-2–associated sepsis vs presumed bacterial sepsis during the first 10 quarters of the COVID-19 pandemic but decreased over time. This finding emphasizes the potential of respiratory viruses to trigger severe, life-threatening disease and underscores the peril of our relative lack of effective antiviral therapeutics for most respiratory viruses. The observed decrease in SARS-CoV-2–associated sepsis mortality overall mirrors other reports demonstrating improving outcomes for patients with COVID-19^35^ and likely reflects a combination of increased immunity from vaccines and prior infections, advances in patient management (antivirals, immunomodulators, increased use of non-invasive respiratory support), and less severe strains on hospital capacity. However, in our analysis of adjusted mortality rates treating quarter as a categorical variable, we observed notably lower mortality during quarter 2, a period with low local SARS-CoV-2 transmission and exceptionally few SARS-CoV-2–associated sepsis cases and deaths, higher mortality during quarter 7 when the Delta variant was locally predominant, followed by a gradual decline beginning in December 2021 when the Omicron variant became predominant. This is consistent with other studies that also found nonlinear improvements, and indeed some increases, in severe COVID-19–associated mortality during the winter 2020 and fall 2021 surges, potentially related to increased virulence of pre-Omicron variants as well as strain on health care systems, despite overall decreases in mortality across the COVID-19 pandemic.^36,37,38^

While distinguishing pure viral sepsis from bacterial sepsis related to a non–SARS-CoV-2 coinfection or superinfection is challenging with an EHR-based approach, only a minority of patients with SARS-CoV-2–associated sepsis had positive blood or sputum cultures identified, supporting SARS-CoV-2 itself as the primary driver of sepsis in most cases. Numerous prior studies have demonstrated that bacterial coinfections are the exception rather than the norm for patients hospitalized with COVID-19.^39,40,41,42^ However, these patients are at high risk for nosocomial bacterial infections, consistent with our observation that many more patients with SARS-CoV-2–associated sepsis had positive cultures across their entire hospitalizations.^17,43^

Our findings may help increase awareness of the underrecognized contribution of viral pathogens to sepsis and to improve nuance in our approach to sepsis diagnosis and treatment. Current sepsis treatment protocols often presume sepsis is caused by bacteria and suggest treating all patients with sepsis with broad-spectrum antibiotics and intravenous fluids. Increasing awareness of nonbacterial sepsis may help reinforce the concept that sepsis is not a one-size-fits-all entity but requires tailoring the diagnostic and therapeutic strategy to each patient’s syndrome and probable pathogen.^44^ Research to develop and evaluate diagnostic tests (particularly for viral pathogens) and biomarkers and/or analyze host-pathogen metagenomics is actively ongoing,^45^ but further study is needed to identify safe and effective strategies for rapidly differentiating sepsis caused by different pathogens to facilitate more targeted treatment and better antibiotic stewardship.

Finally, while this analysis was limited to a comparison of sepsis associated with SARS-CoV-2 infection vs presumed bacterial sepsis, other viruses, such as influenza A, influenza B, and RSV, can also cause severe disease that is consistent with viral sepsis. The approach outlined in this study may be useful to define the burden and outcomes for other causes of respiratory viral sepsis, particularly during seasonal surges.

### Limitations

This study has limitations. First, it was performed in a single health system; further study using geographically and demographically diverse data sets is needed. Second, while the EHR-based criteria performed well compared with in-depth medical record review, the capture of sepsis cases using an EHR-based approach is imperfect. In particular, identification of SARS-CoV-2–associated sepsis is contingent upon testing for the SARS-CoV-2 virus, and thus estimates of prevalence and mortality are subject to ascertainment bias as testing rates change based upon clinician behavior, hospital policies, and public health recommendations. In hospital systems with widespread use of SARS-CoV-2 testing for all inpatients, such as MGB, PPV may be lower than in systems that perform more targeted testing because the chance of diagnosing incidental or noncontributing SARS-CoV-2 infections rises with increased testing.^46^ Third, we may have misattributed organ dysfunction to SARS-CoV-2, particularly during periods of high community transmission when some hospitalized patients tested positive for SARS-CoV-2 while hospitalized for other reasons.^22^ Fourth, our analyses of mortality trends for presumed bacterial sepsis may have been confounded by changes in case mix for the non–COVID-19 population over time. We did adjust our estimates for patient demographics and comorbidities, but we did not adjust for vital signs or laboratory measures, allowing for the possibility of residual confounding. Fifth, there were substantial fluctuations in quarterly incidence and in-hospital mortality for SARS-CoV-2–associated sepsis, and therefore the linear models and reported IRRs are imperfect representations of pandemic trends. We chose to use linear models to provide an easily understandable quantification of the overall declines seen qualitatively in both linear and nonlinear approaches as well as to facilitate comparison of trends for SARS-CoV-2–associated sepsis and presumed bacterial sepsis.

## Conclusions

SARS-CoV-2 accounted for approximately 1 in 6 cases of sepsis during the first 33 months of the COVID-19 pandemic. In-hospital mortality rates for SARS-CoV-2–associated sepsis were high but declined over time and became comparable with presumed bacterial sepsis. These findings highlight the high burden of SARS-CoV-2–associated sepsis and demonstrate the feasibility of using EHR-based algorithms to conduct surveillance for viral sepsis.

## Notes

Supplement 1. **eTable 1.** Performance of Alternate EHR-Based Definitions of SARS-CoV-2–Associated Sepsis vs Physician Review **eTable 2.** Summary of SARS-CoV-2–Associated and Presumed Bacterial Sepsis Encounters and Deaths by Quarter **eTable 3.** Results of Sensitivity Analyses: Poisson and Logistic Regression Models for Mortality With Alternative Definitions of SARS-CoV-2–Associated Sepsis **eFigure 1.** Distribution and Number of Organ Dysfunction Categories for SARS-CoV-2 and Presumed Bacterial Sepsis **eFigure 2.** Incidence and Mortality for SARS-CoV-2–Associated and Presumed Bacterial Sepsis by Quarter

Supplement 2.
Data Sharing Statement
