# Supplementary material for: Use of Electronic Clinical Data to Track Incidence and Mortality for SARS-CoV-2–Associated Sepsis
Source: JAMA Netw Open. 2023 Sep 29;6(9):e2335728. doi: 10.1001/jamanetworkopen.2023.35728 (PMC10543118; doi:10.1001/jamanetworkopen.2023.35728)
Supplement: Supplement 1. — eTable 1. Performance of Alternate EHR-Based Definitions of SARS-CoV-2–Associated Sepsis vs Physician Review eTable 2. Summary of SARS-CoV-2–Associated and Presumed Bacterial Sepsis Encounters and Deaths by Quarter eTable 3. Results of Sensitivity Analyses: Poisson and Logistic Regression Models for Mortality With Alternative Definitions of SARS-CoV-2–Associated Sepsis eFigure 1. Distribution and Number of Organ Dysfunction Categories for SARS-CoV-2 and Presumed Bacterial Sepsis eFigure 2. Incidence and Mortality for SARS-CoV-2–Associated and Presumed Bacterial Sepsis by Quarter [file jamanetwopen-e2335728-s001.pdf]

Supplemental Online Content

Shappell CN, Klompas M, Chan C, et al. Electronic clinical data to track incidence and mortality for SARS-CoV-2–associated sepsis. *JAMA Netw Open*. 2023;6(9):e2335728. doi:10.1001/jamanetworkopen.2023.35728

eTable 1. Performance of Alternate EHR-Based Definitions of SARS-CoV-2–Associated Sepsis vs Physician Review

eTable 2. Summary of SARS-CoV-2–Associated and Presumed Bacterial Sepsis Encounters and Deaths by Quarter

eTable 3. Results of Sensitivity Analyses: Poisson and Logistic Regression Models for Mortality With Alternative Definitions of SARS-CoV-2–Associated Sepsis

eFigure 1. Distribution and Number of Organ Dysfunction Categories for SARS-CoV-2 and Presumed Bacterial Sepsis

eFigure 2. Incidence and Mortality for SARS-CoV-2–Associated and Presumed Bacterial Sepsis by Quarter

This supplemental material has been provided by the authors to give readers additional information about their work.

© 2023 Shappell CN et al. *JAMA Network Open*.

eTable 1. Performance of Alternate EHR-based Definitions of SARS-CoV-2-associated Sepsis vs Physician Review

Alternate Definition	Sensitivity % (95% CI)	Specificity % (95% CI)	AUROC (95% CI)	PPV % (95% CI)	NPV % (95% CI)
**Original***	90.6 (80.7-96.5)	91.2 (85.1-95.4)	0.91 (0.87-0.95)	82.9 (72.0-90.8)	95.4 (90.2-98.3)
**Narrower Window for Organ Dysfunction (7 days)**	89.1 (78.8-95.5)	91.2 (85.1-95.4)	0.90 (0.86-0.95)	82.6 (71.6-90.7)	94.7 (89.3-97.8)
**SARS-CoV-2 Encounter identified with ICD-10 codes**	89.1 (78.8-95.5)	90.4 (84.2-94.8)	0.90 (0.85-0.94)	81.4 (70.3-89.7)	94.6 (89.2-97.8)
**eSOFA Respiratory Dysfunction requiring MV, HFNC, or NIPPV**	75.0 (62.6-85.0)	92.6 (86.9-96.4)	0.84 (0.78-0.90)	82.8 (70.6-91.4)	88.7 (82.3-93.4)

*Positive SARS-CoV-2 PCR or institutional COVID flag to identify SARS-CoV-2 encounter, 14 day window for organ dysfunction, eSOFA respiratory dysfunction equivalent to any oxygen requirement greater than simple nasal cannula; see Box for full criteria

© 2023 Shappell CN et al. *JAMA Network Open*.

eTable 2. Summary of SARS-CoV-2-associated and Presumed Bacterial Sepsis Encounters and Deaths by Quarter

Quarter	All Encounters	SARS-CoV-2			Presumed Bacterial	
		Encounters	Sepsis Encounters n (%)	Sepsis Deaths n (%)	Sepsis Encounters n (%)	Sepsis Deaths n (%)
1	29,615	3,704	1,469 (5.0)	490 (33.4)	2,204 (7.4)	307 (13.9)
2	38,361	655	157 (0.4)	20 (12.7)	2,889 (7.5)	419 (14.5)
3	39,086	1,343	350 (0.9)	90 (25.7)	3,036 (7.8)	420 (13.8)
4	35,981	3,775	1,111 (3.1)	243 (21.9)	2,670 (7.4)	395 (14.8)
5	41,764	1,415	469 (1.1)	79 (16.8)	2,867 (6.9)	387 (13.5)
6	43,012	690	212 (0.5)	43 (20.3)	3,179 (7.4)	431 (13.6)
7	41,068	1,250	403 (1.0)	100 (24.8)	2,913 (7.1)	440 (15.1)
8	37,176	4,285	1,145 (3.1)	218 (19.0)	2,514 (6.8)	409 (16.3)
9	42,029	1,756	363 (0.9)	55 (15.2)	2,791 (6.6)	443 (15.9)
10	41,986	2,226	429 (1.0)	55 (12.8)	2,866 (6.8)	438 (15.3)
11	40,939	2,177	450 (1.1)	67 (14.9)	2,675 (6.5)	362 (13.5)
Total	431,017	23,276	6,558 (1.5)	1,460 (22.3)	30,604 (7.1)	4,451 (14.5)

© 2023 Shappell CN et al. *JAMA Network Open*.

eTable 3. Results of sensitivity analyses: Poisson and Logistic Regression models for mortality with alternative definitions of SARS-CoV-2-associated sepsis

Alternative Definition	**Unadjusted Mortality by Quarter** *Negative Binomial Regression*		**Adjusted Mortality by Quarter (continuous)** *Logistic Regression*	
	IRR[a]	95% CI[b]	OR[c]	95% CI[b]
**Narrower Window for Organ Dysfunction (7 days)**	0.94	0.90 – 0.97	0.88	0.86 – 0.90
**SARS-CoV-2 Encounter identified with ICD-10 code**	0.94	0.90 – 0.98	0.89	0.87 – 0.91
**eSOFA Respiratory Dysfunction requiring MV, HFNC, or NIPPV**	0.96	0.93 – 0.98	0.91	0.89 – 0.93

[a]IRR= incidence rate ratio, [b]CI= confidence interval, [c]OR=odds ratio

© 2023 Shappell CN et al. *JAMA Network Open*.

eFigure 1. Distribution and Number of Organ Dysfunction Categories for SARS-CoV-2 and Presumed Bacterial Sepsis

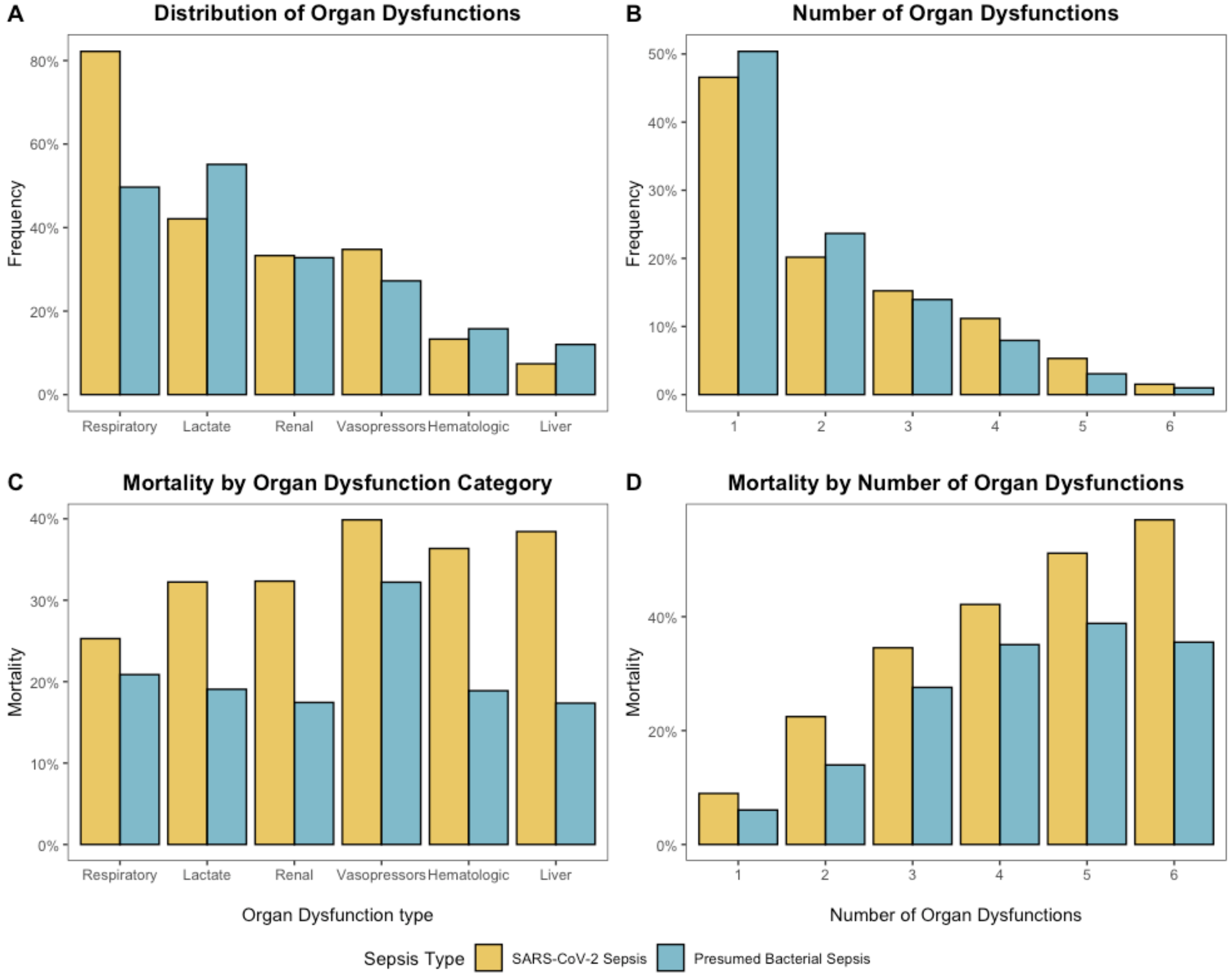

© 2023 Shappell CN et al. *JAMA Network Open*.

eFigure 2. Incidence and Mortality for SARS-CoV-2-Associated and Presumed Bacterial Sepsis by Quarter

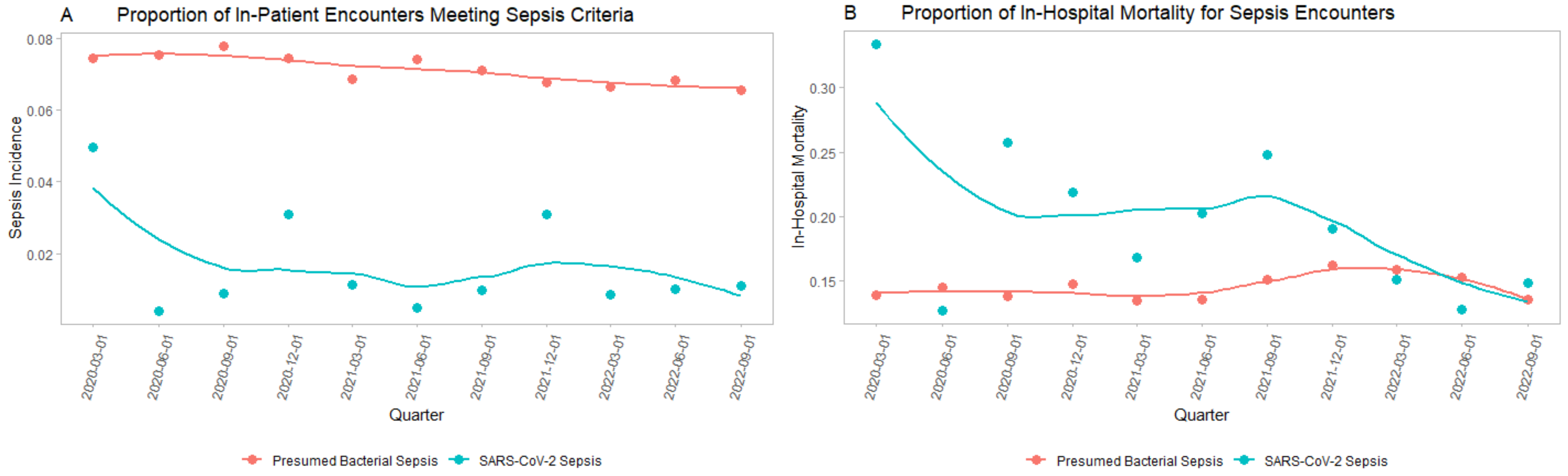

© 2023 Shappell CN et al. *JAMA Network Open*.
