# Supplementary material for: Use of Electronic Clinical Data to Track Incidence and Mortality for SARS-CoV-2–Associated Sepsis
Source: JAMA Netw Open. 2023 Sep 29;6(9):e2335728. doi: 10.1001/jamanetworkopen.2023.35728 (PMC10543118; doi:10.1001/jamanetworkopen.2023.35728)
Supplement: Supplement 2. — Data Sharing Statement [file jamanetwopen-e2335728-s002.pdf]

Data Sharing Statement

Data
Data available: No

Additional Information
Explanation for why data not available: De-identified patient data will be made available by reasonable request to the corresponding author, subject to MassGeneralBrigham data sharing policy, institutional review board approval, and processing fees. Statistical/analytic code will be shared freely by request.
